# Supplementary material for: Endometriotic lesions exhibit distinct metabolic signature compared to paired eutopic endometrium at the single-cell level
Source: Commun Biol. 2024 Aug 21;7:1026. doi: 10.1038/s42003-024-06713-5 (PMC11339455; doi:10.1038/s42003-024-06713-5)
Supplement: Supplementary file 2 — Supplementary Information [file 42003_2024_6713_MOESM2_ESM.pdf]

Supplementary Information for manuscript: "Endometriotic lesions exhibit distinct metabolic signature compared to paired eutopic endometrium at the single-cell level"

Authors: Meruert Sarsenova[a,b,c,d], Ankita Lawarde[a,d], Amruta D. S. Pathare[a,d], Merli Saare[a,d], Vijayachitra Modhukur[a,d], Pille Soplepmann[e], Anton Terasmaa[f], Tuuli Käämbre[f], Kristina Gemzell-Danielsson[b,c], Parameswaran Grace Luther Lalitkumar[b,c], Andres Salumets[a,d,g,h*], Maire Peters[a,d]

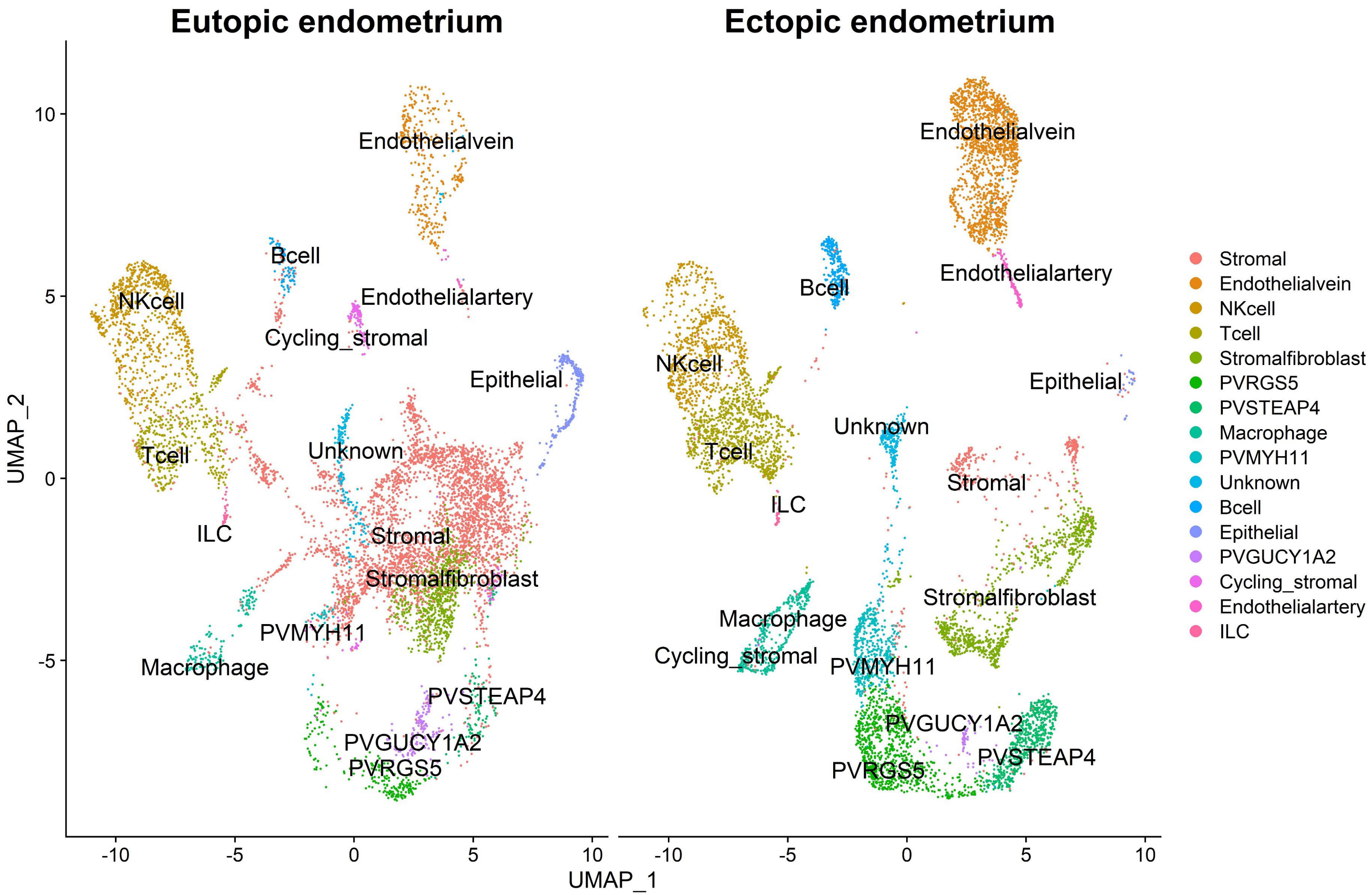

Supplementary Figure 1. UMAP of 16 unmerged clusters. The clusters of eutopic endometrium – on the left side, ectopic endometrium – on the right side.

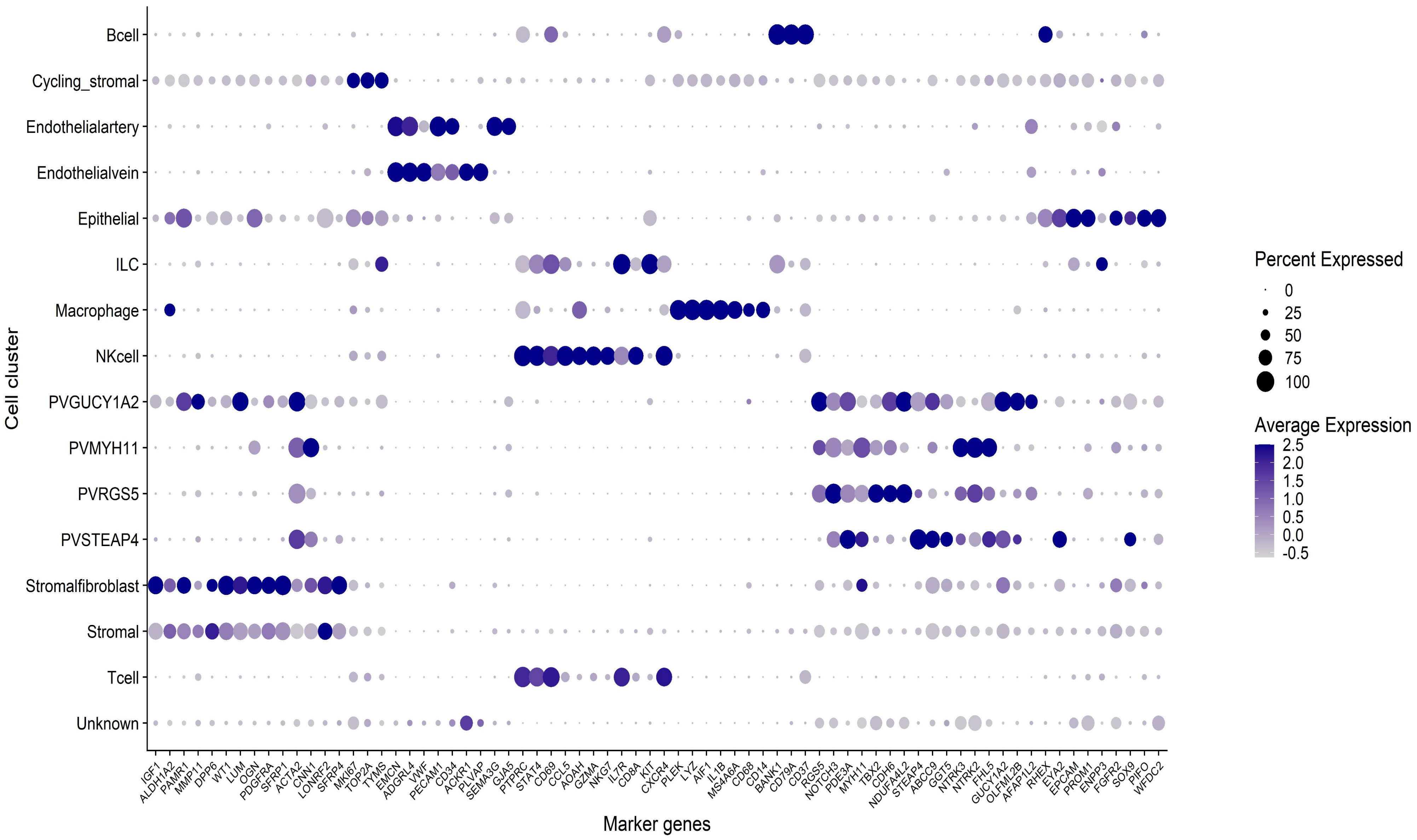

Supplementary Figure 2. Differentially expressed gene markers in 16 unmerged clusters. Each dot shows the average gene expression and percent expressed. Each cluster exhibited the expression of cell-type representative marker genes.

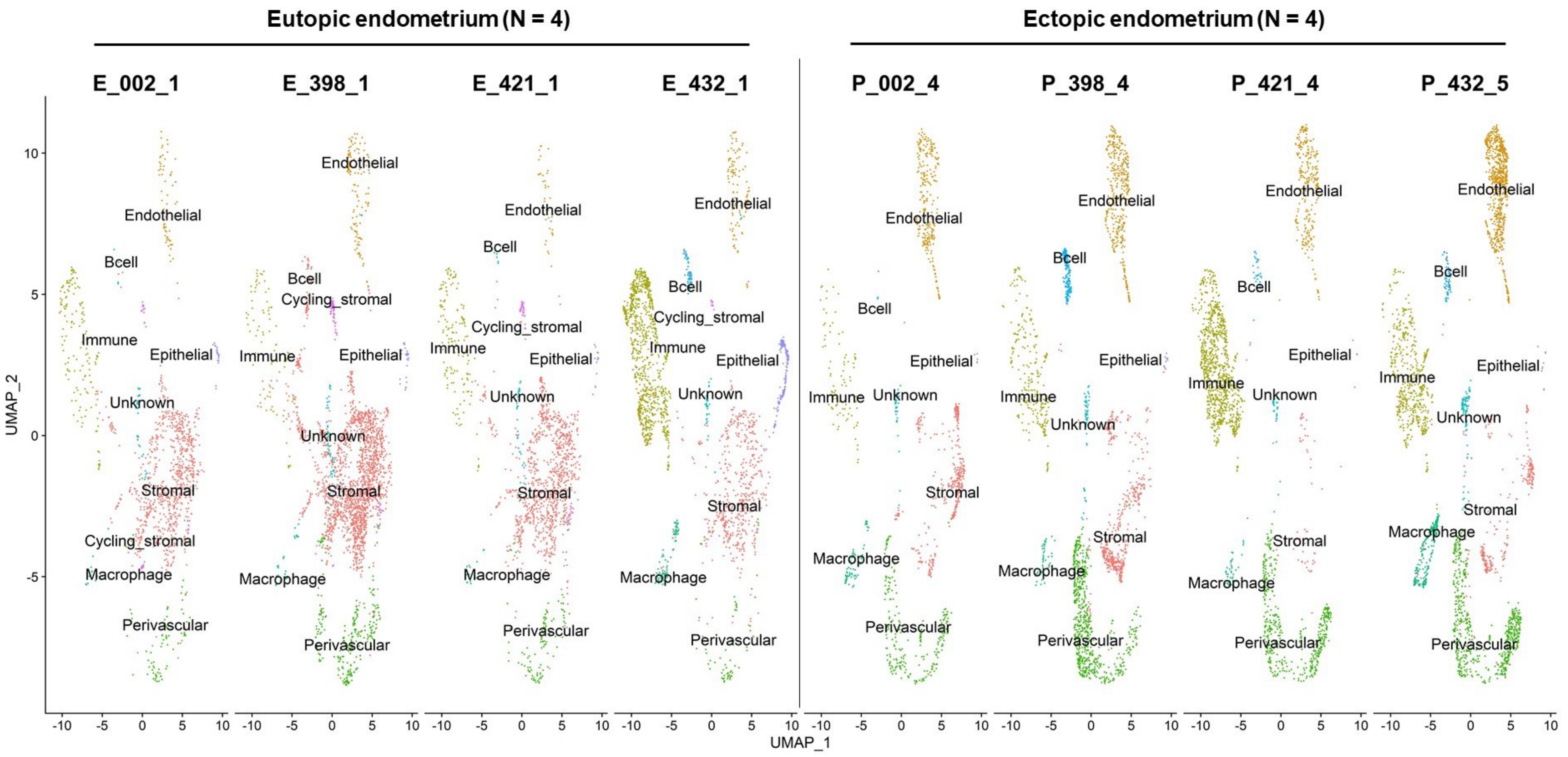

Supplementary Figure 3. UMAP of 9 major clusters in 8 samples (4 EuE and 4 EcE) showing inter-patient similarity within the two groups (EuE or EcE) and inter-group variability. The clusters of eutopic endometria – on the left side, ectopic endometria – on the right side.

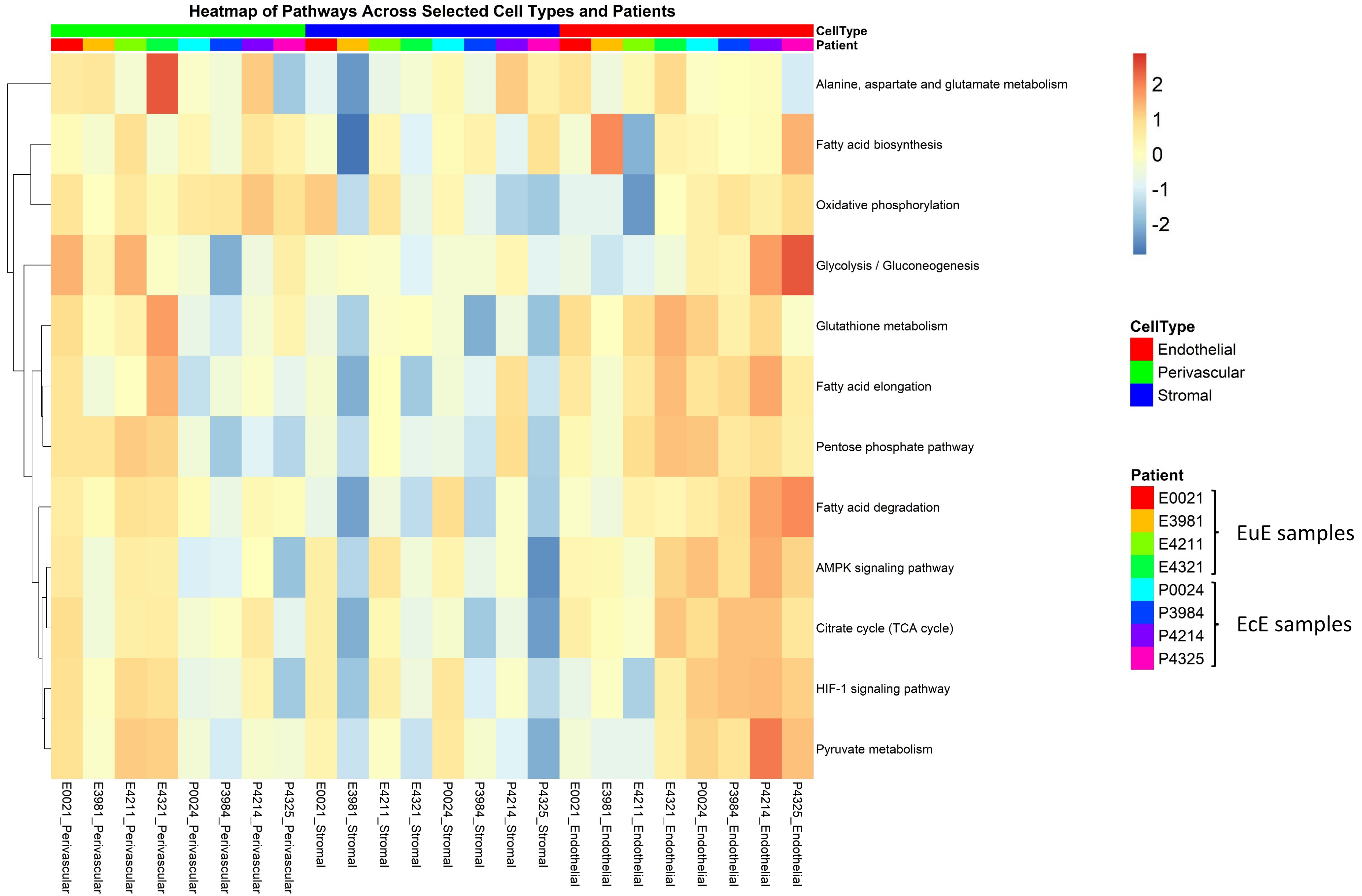

Supplementary Figure 4. Heatmap showing the metabolic pathway (N = 12) activity in perivascular, stromal and endothelial cell populations in each sample. EuE – eutopic endometrium; EcE – ectopic endometrium; the GSVA score ranging from blue to red corresponds to sample-wise pathway activity.

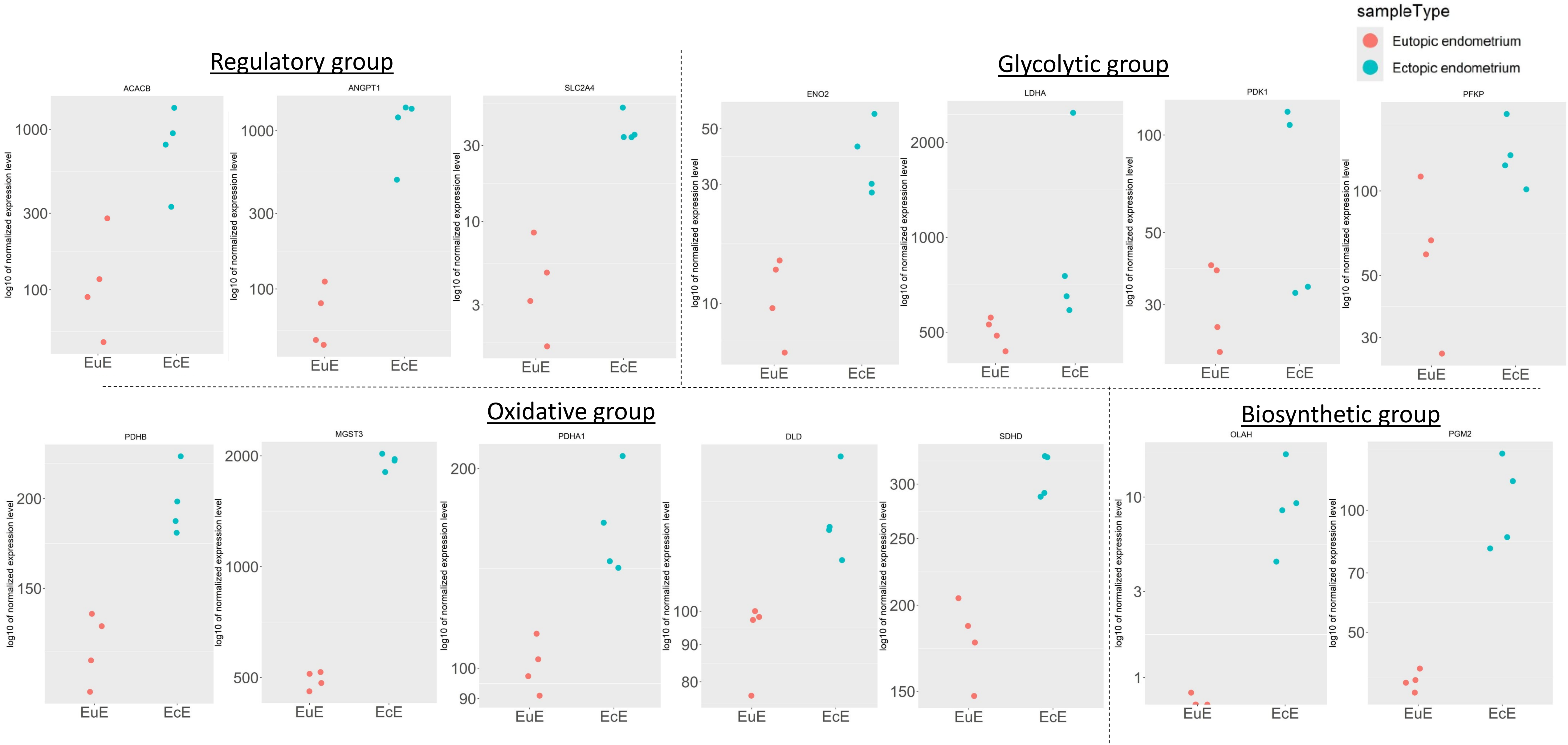

Supplementary Figure 5. Dot-plots of representative metabolic DEGs ($p_{adj} < 0.05$, log_{10} of normalised expression level) from four main metabolic groups (Regulatory pathways, Glycolytic metabolism, Oxidative metabolism and Biosynthetic pathways, from Table 2 in the manuscript), illustrating the level of expression in **perivascular** cells across eutopic (EuE, N = 4, red dots) and ectopic (EcE, N = 4, blue dots) endometrial samples.

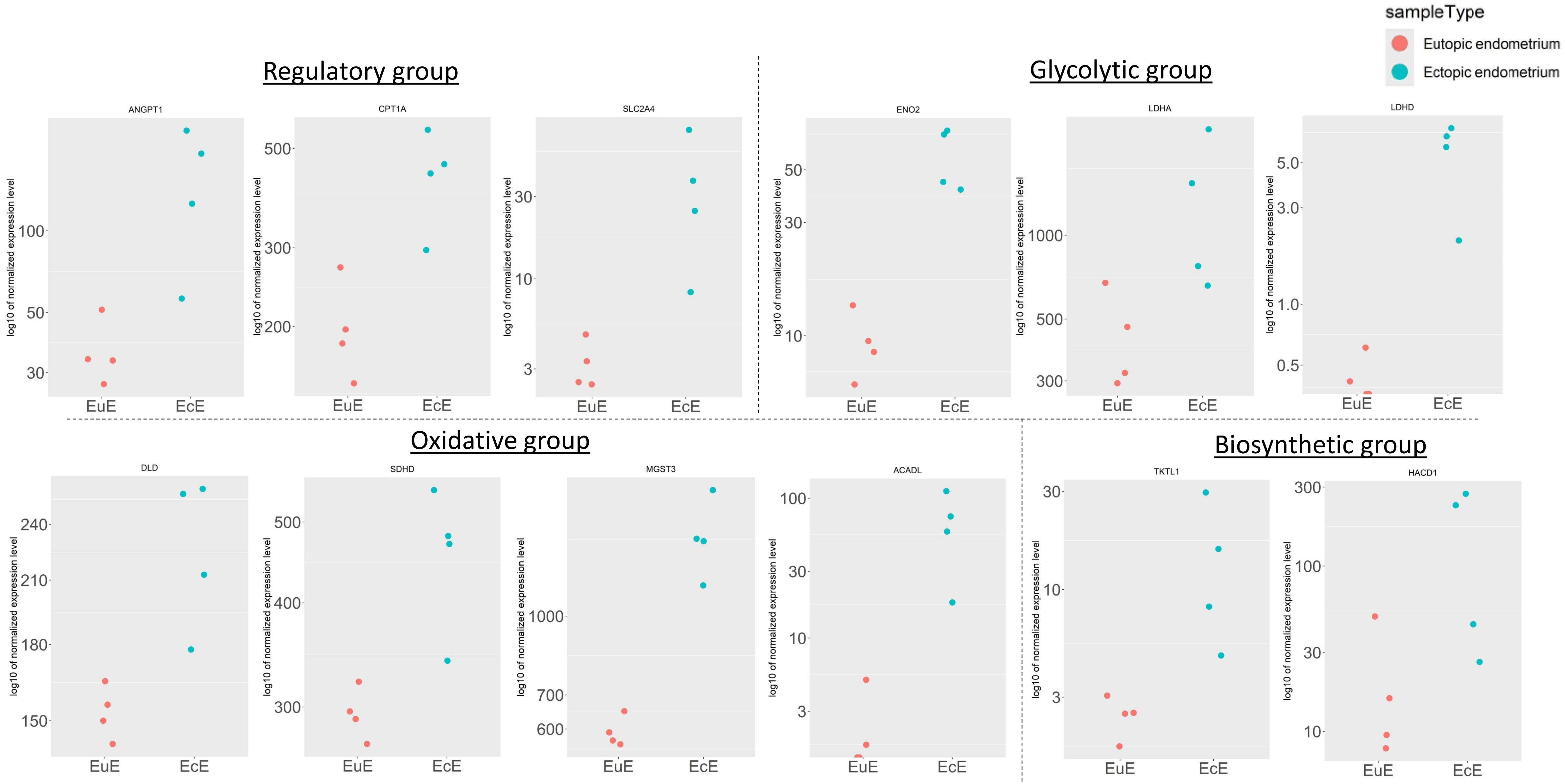

Supplementary Figure 6. Dot-plots of representative metabolic DEGs ($p_{adj} < 0.05$, log_{10} of normalised expression level) from four main metabolic groups (Regulatory pathways, Glycolytic metabolism, Oxidative metabolism and Biosynthetic pathways, from Table 2 in the manuscript), illustrating the level of expression in **stromal** cells across eutopic (EuE, N = 4, red dots) and ectopic (EcE, N = 4, blue dots) endometrial samples.

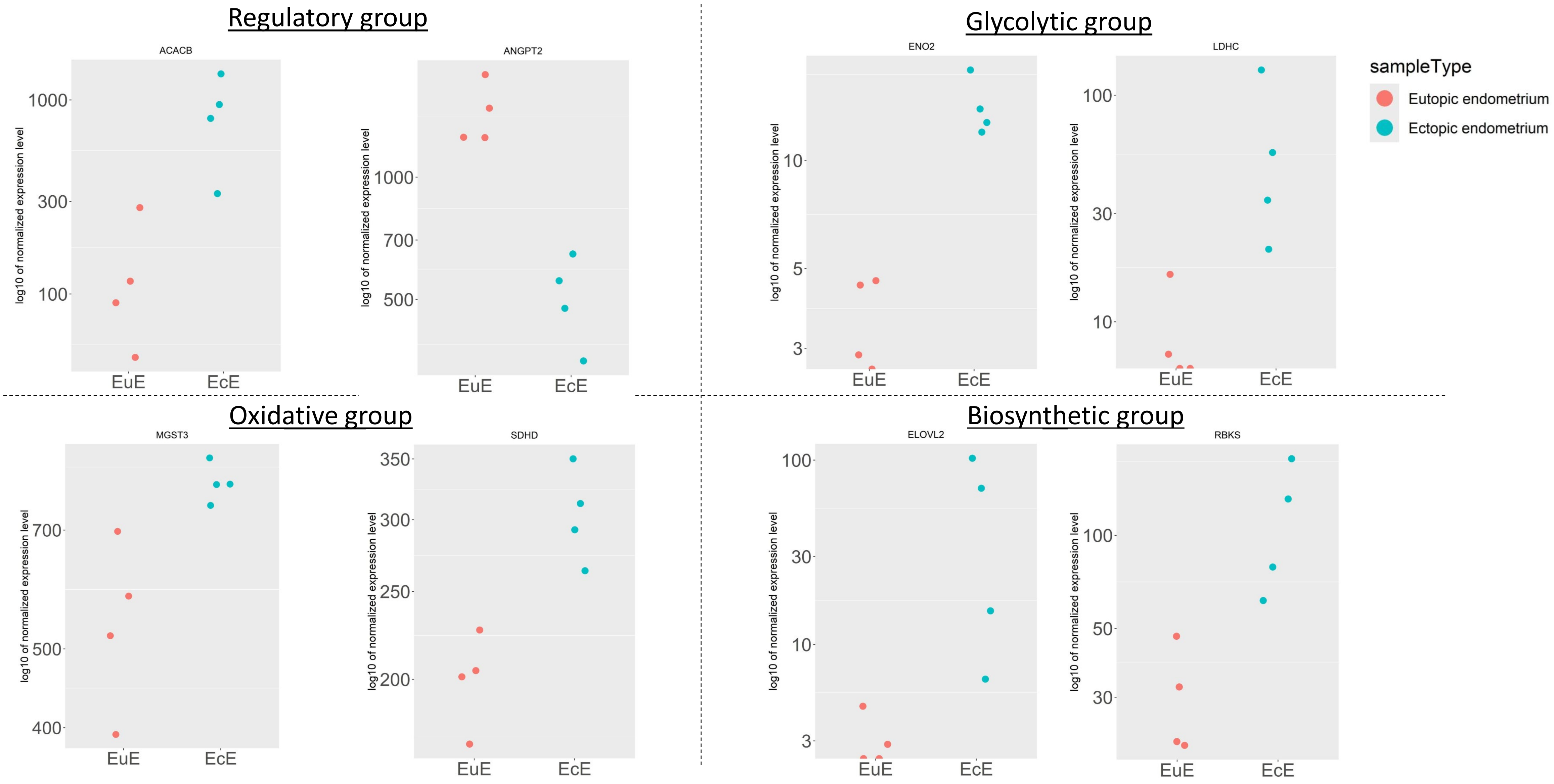

Supplementary Figure 7. Dot-plots of representative metabolic DEGs ($p_{adj} < 0.05$, log_{10} of normalised expression level) from four main metabolic groups (Regulatory pathways, Glycolytic metabolism, Oxidative metabolism and Biosynthetic pathways, from Table 2 in the manuscript), illustrating the level of expression in **endothelial** cells across eutopic (EuE, N = 4, red dots) and ectopic (EcE, N = 4, blue dots) endometrial samples.

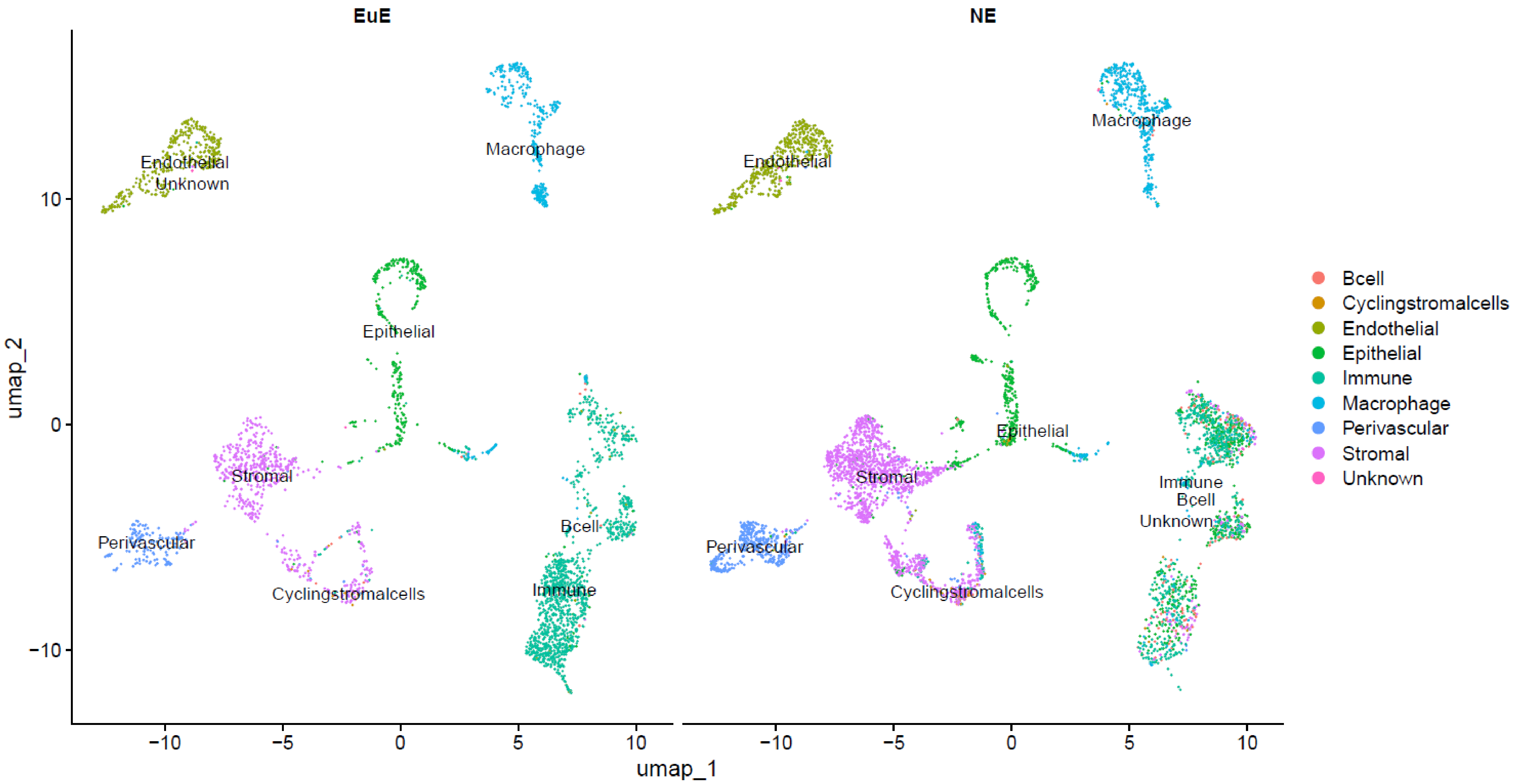

Supplementary Figure 8. UMAP of 9 major clusters identified in the eutopic endometrium (EuE, on the left side) and controls (NE, on the right side) from the external dataset (Huang et al. 2023).

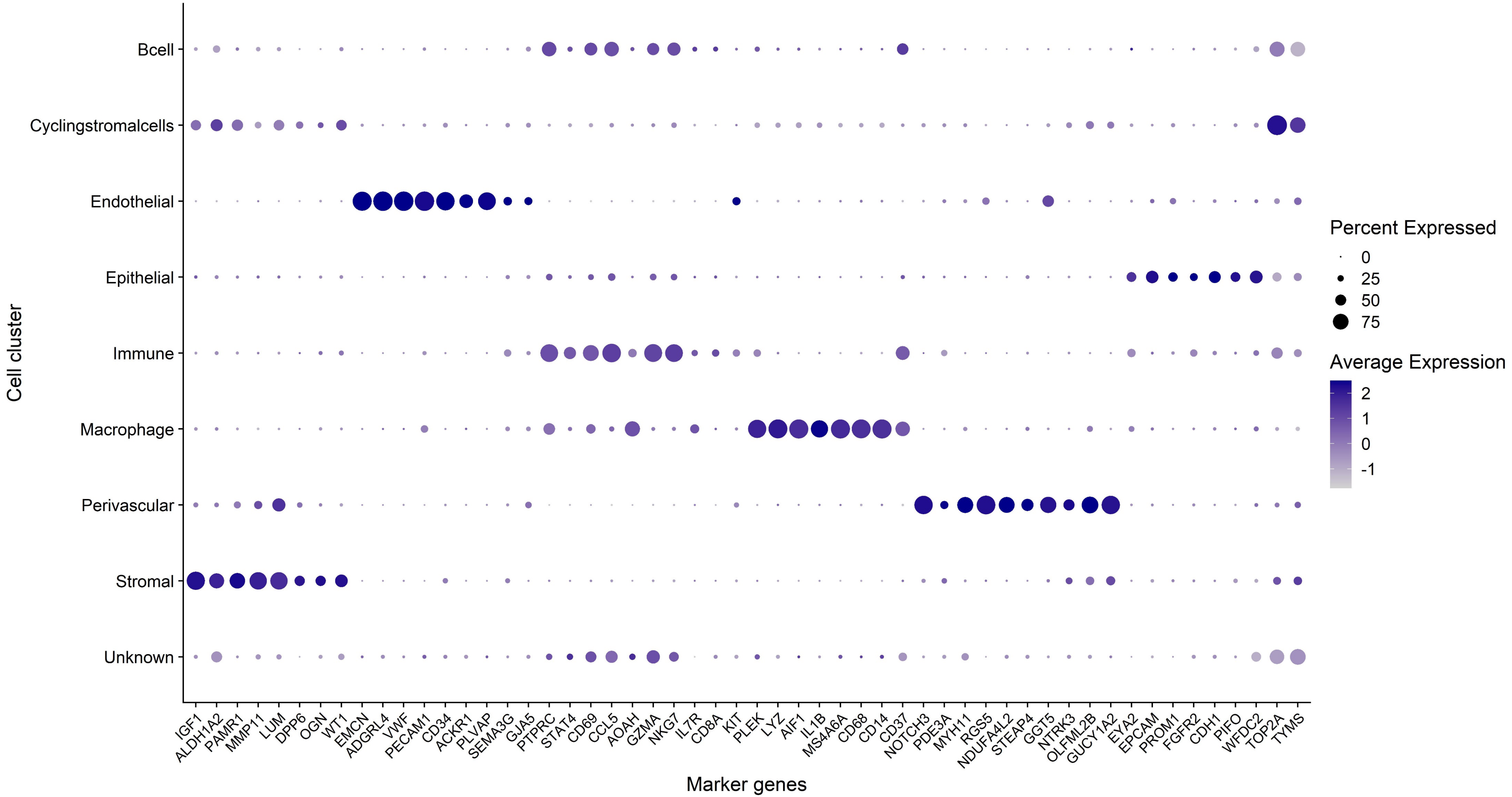

Supplementary Figure 9. Differentially expressed gene markers in 9 major clusters identified in the eutopic endometrium from women with endometriosis and controls from the external dataset (Huang et al. 2023). Each dot shows the average gene expression and percent expressed. Each cluster exhibited the expression of cell-type representative marker genes.

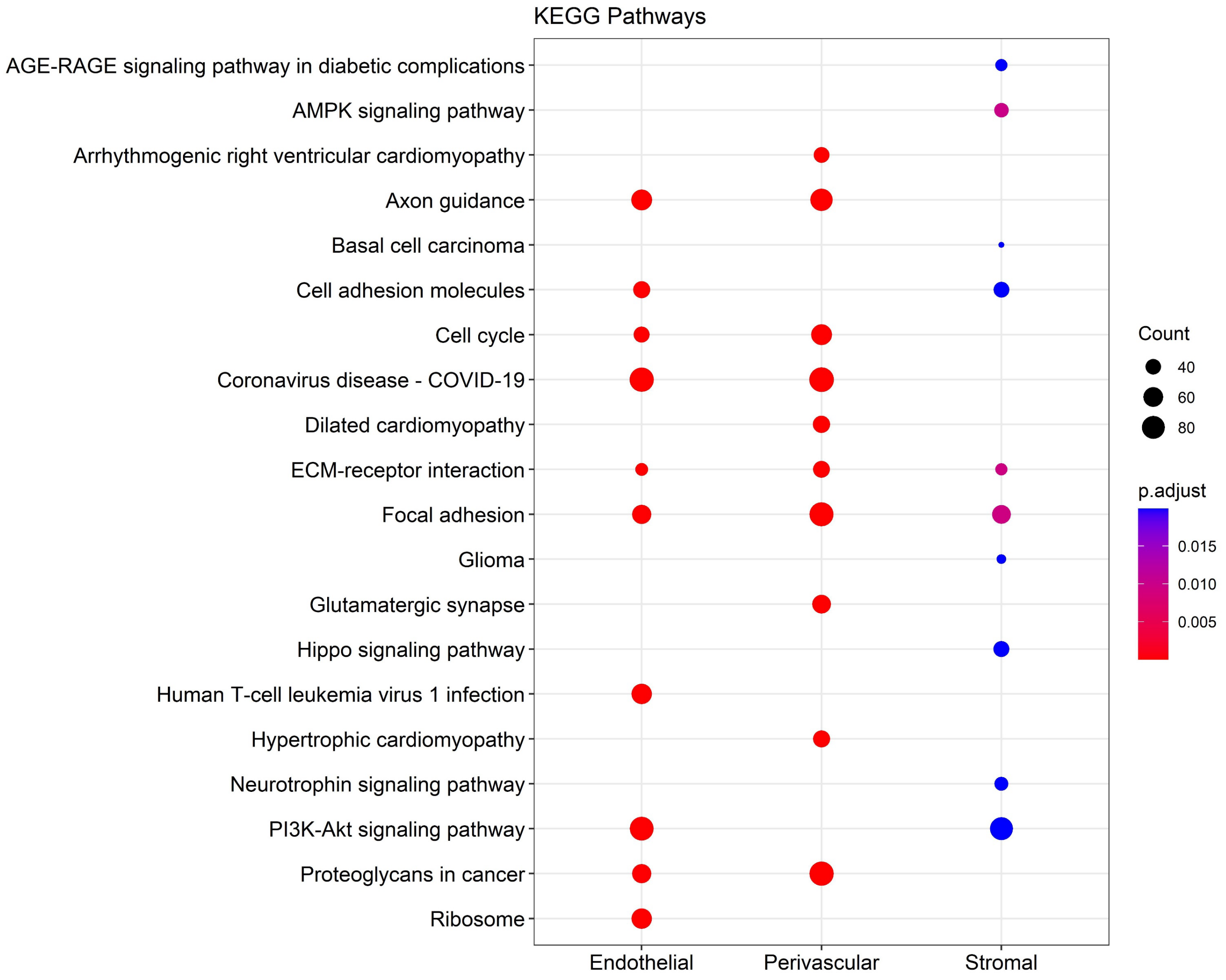

Supplementary Figure 10. Top 10 enriched KEGG pathways in perivascular, stromal and endothelial cell populations of ectopic endometrium compared to eutopic endometrium.

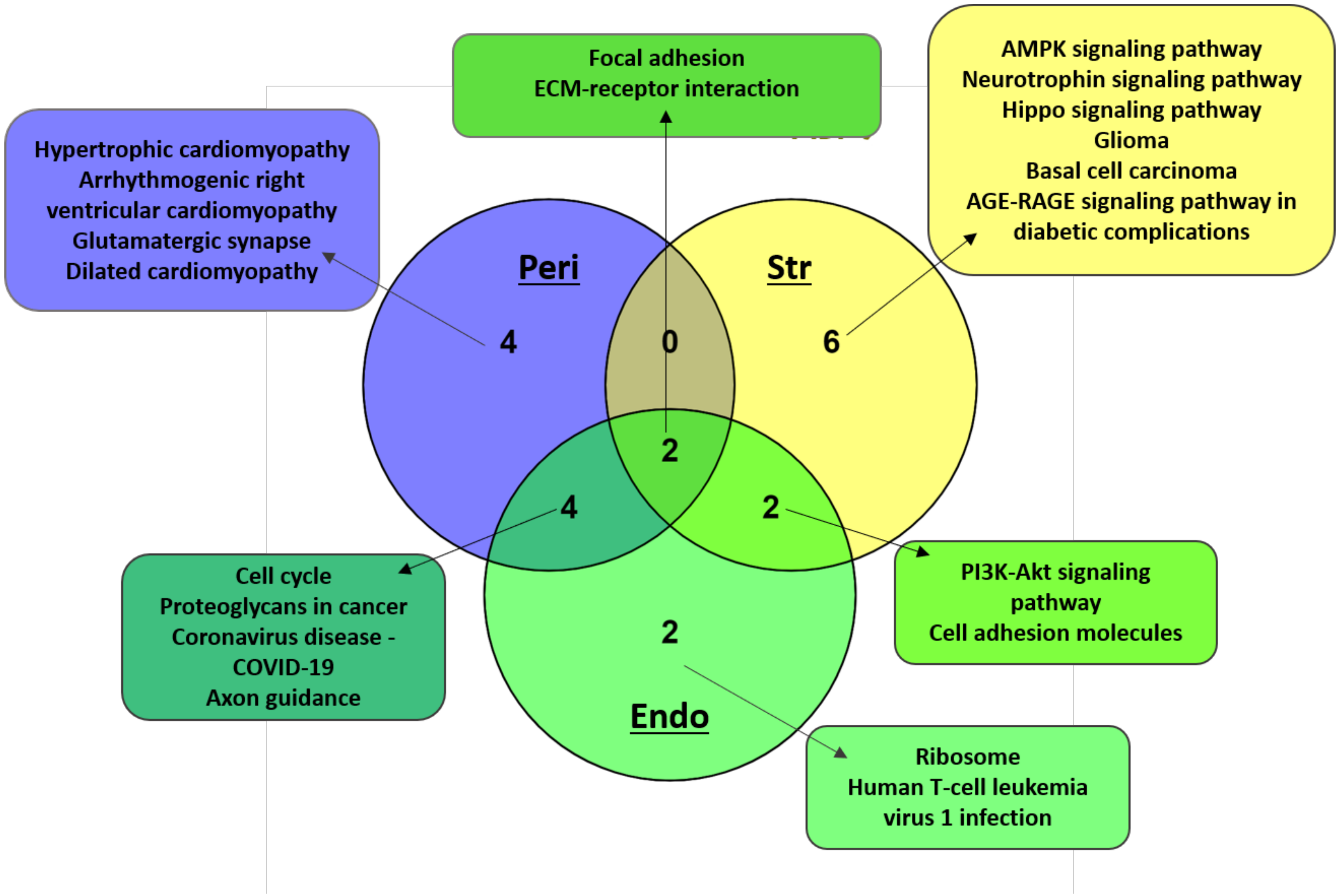

Supplementary Figure 11. Venn diagram of top 10 enriched KEGG pathways in perivascular, stromal and endothelial cell populations of ectopic endometrium compared to eutopic endometrium.

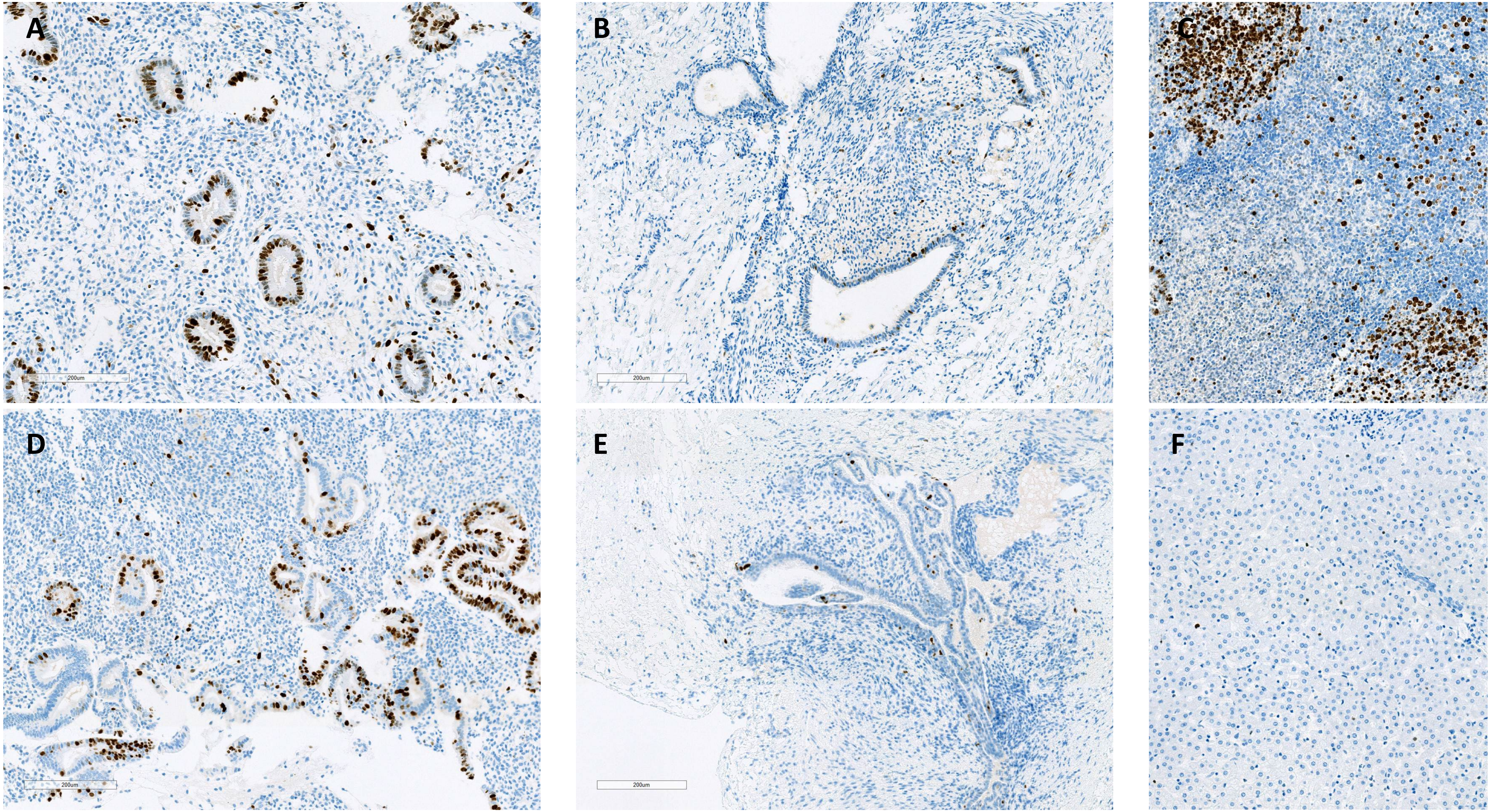

Supplementary Figure 12. Representative images of Ki67 staining of eutopic endometrium (A, D) and ectopic endometrium (peritoneal lesion, B, E) from patients with endometriosis, showing higher expression of Ki67 proliferative marker in glands and stroma of eutopic endometrium compared to ectopic endometrium. Positive control (C, tonsil stroma) and a negative control (F, liver) for Ki67 staining. Scale bar 200 μm.

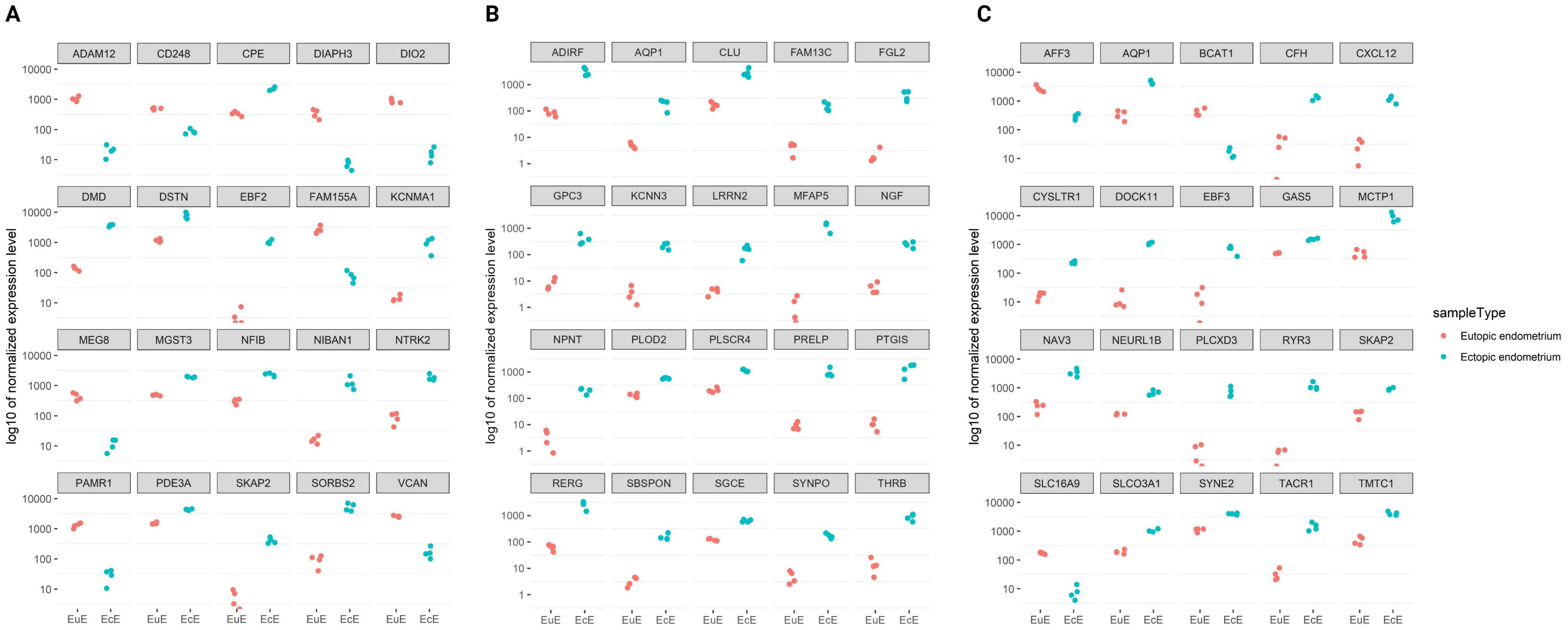

Supplementary Figure 13. Top 20 DEGs ($p_{adj} < 0.05$, $\log_2$ of normalised expression level) of perivascular (A), stromal (B) and endothelial (C) populations. The samples of Eutopic endometrium (EuE) – in red, ectopic endometrium (EcE) – in blue.

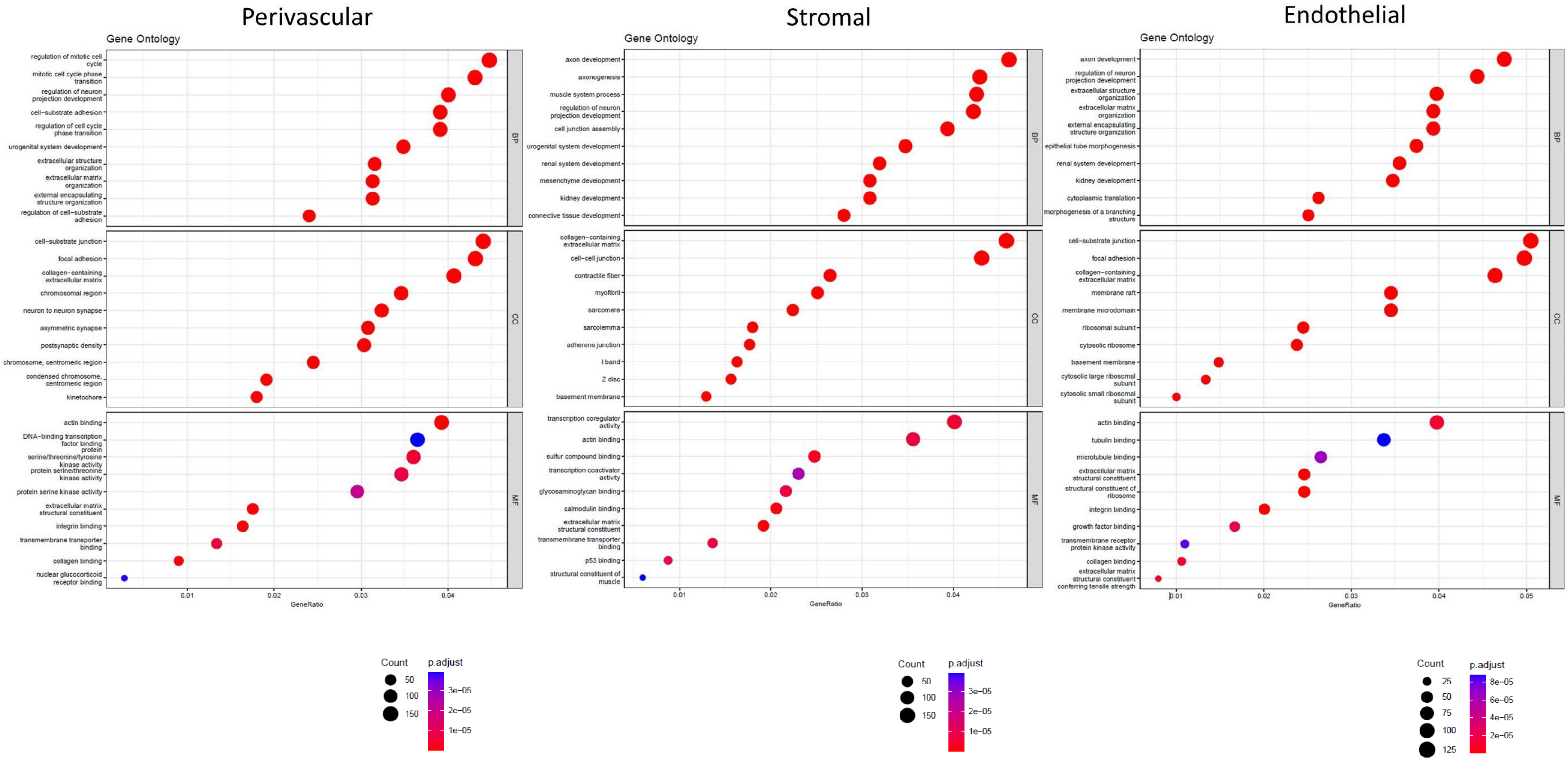

Supplementary Figure 14. GO enrichment analysis with the top 10 biological process (BP), molecular function (MF) and cell compartment (CC) for perivascular, stromal and endothelial cell types.

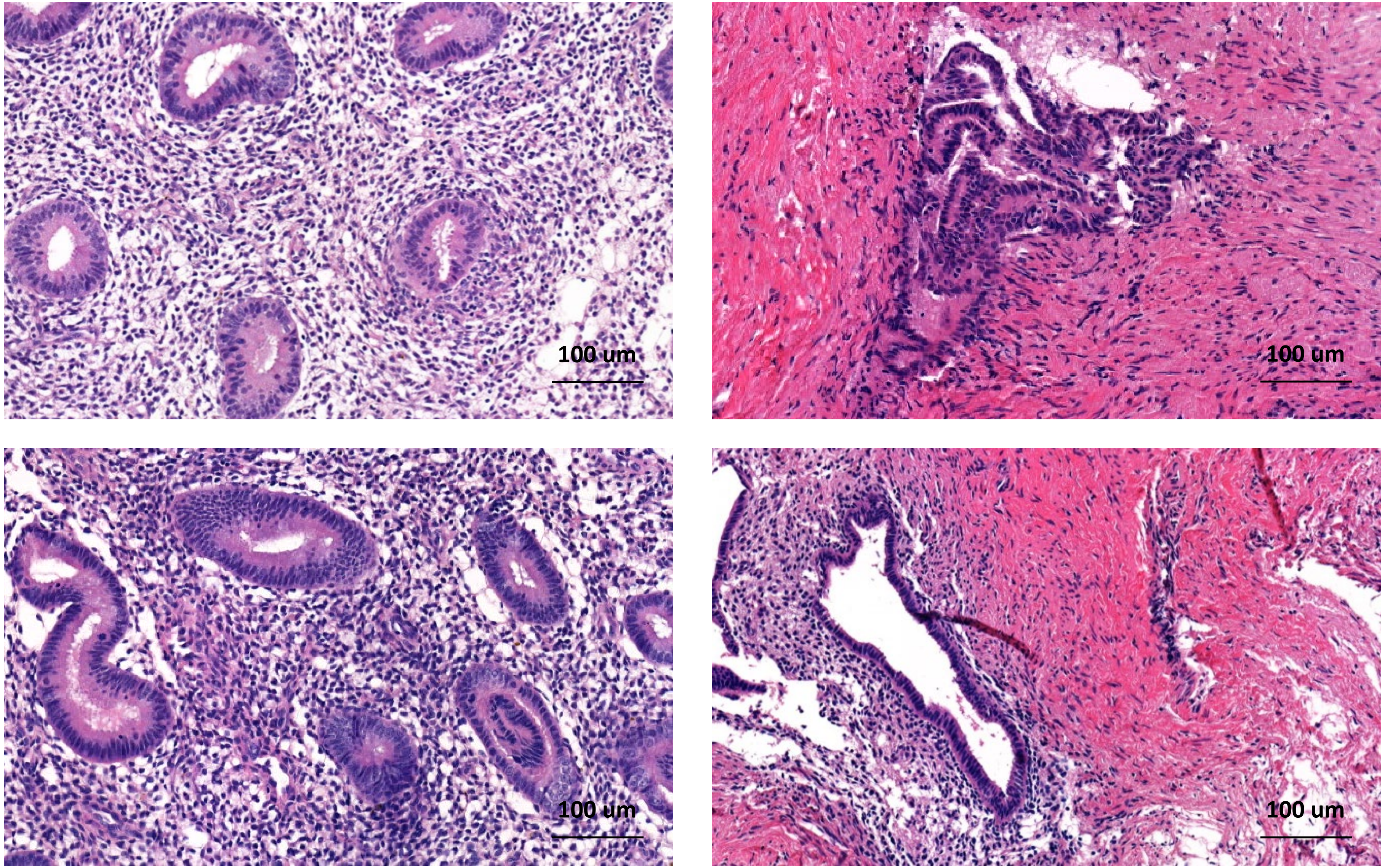

Supplementary Figure 15. Representative images of haematoxylin and eosin staining of eutopic endometrium (left images) and ectopic endometrium (peritoneal lesion, right images) from two patients (upper images and lower images) with endometriosis, showing morphological differences between paired eutopic and ectopic endometrial tissues. Scale bar 100 μm.

Supplementary Table 1. Total cell counts and percentages for all clusters in ectopic endometrium (EcE) and eutopic endometrium (EuE). Fisher's exact test was used to compare cell cluster proportions between EcE and EuE.

Cluster, cell count (%)	Stromal	Endothelial	Immune	Perivascular	Macrophage
EcE	1248 (16.6%)	1630 (21.6%)	1785 (23.7%)	1959 (26.0%)	380 (5.0%)
EuE	4284 (58.9%)	335 (4.6%)	1314 (18.2%)	527 (7.2%)	143 (2.0 %)
Fisher's exact (P value)	<1.13E-103	<1.13E-103	5.56E-33	<1.13E-103	5.93E-50

Cluster, cell count (%)	Unknown	B cell	Epithelial	Cycling stromal	Total count
EcE	230 (3.1%)	281 (3.7%)	22 (0.3%)	3 (0%)	7538
EuE	177 (2.4%)	67 (0.9%)	241 (3.3%)	191 (2.6%)	7279
Fisher's exact (P value)	0.000262437	7.39E-63	1.03E-93	1.13E-103	

Supplementary Table 2. Analysis of differential expression of 12 metabolic pathways between ectopic endometrium (EcE) and eutopic endometrium (EuE). Numbers of differentially expressed genes of metabolic pathways and percentages of the genes calculated to the total N of genes in a pathway.

Metabolic Pathways	Total N of genes in a pathway	Perivascular, N (%)	Stromal, N (%)	Endothelial, N (%)	Immune, N (%)
ALA, AST and GLU metabolism	37	9 (24.3%)	2 (5.4%)	7 (18.9%)	0
AMPK signaling pathway	121	39 (32.2%)	35 (28.9%)	23 (19.0%)	3 (2.5%)
Citrate cycle (TCA cycle)	30	10 (33.3%)	5 (16.7%)	3 (10.0%)	0
Fatty acid biosynthesis	18	4 (22.2%)	1 (5.6%)	3 (16.7%)	0
Fatty acid degradation	43	18 (41.9%)	10 (23.3%)	2 (4.7%)	0
Fatty acid elongation	27	12 (44.4%)	7 (25.9%)	3 (11.1%)	0
Glutathione metabolism	57	16 (28.1%)	13 (22.8%)	12 (21.1%)	2 (3.5%)
Glycolysis / Gluconeogenesis	67	21 (31.3%)	12 (17.9%)	7 (10.4%)	1 (1.5%)
HIF-1 signaling pathway	109	39 (35.8%)	22 (20.2%)	21 (19.3%)	6 (5.5%)
Oxidative phosphorylation	134	32 (23.9%)	10 (7.5%)	5 (3.7%)	0
Pentose phosphate pathway	30	11 (36.7%)	5 (16.7%)	3 (10.0%)	0
Pyruvate metabolism	47	16 (34.0%)	12 (25.5%)	5 (10.6%)	0

Metabolic Pathways	Total N of genes in a pathway	Cycling stromal, N (%)	Epithelial, N (%)	Unknown, N (%)	Macrophages, N (%)	B cells, N (%)
ALA, AST and GLU metabolism	37	0	1 (2.7%)	2 (5.4%)	0	0
AMPK signaling pathway	121	6 (5%)	0	4 (3.3%)	0	0
Citrate cycle (TCA cycle)	30	0	0	1 (3.3%)	0	0
Fatty acid biosynthesis	18	0	0	1 (5.6%)	0	0
Fatty acid degradation	43	0	0	0	0	0
Fatty acid elongation	27	0	0	0	0	0

Glutathione metabolism	57	1 (1.8%)	0	4 (7%)	0	0
Glycolysis / Gluconeogenesis	67	0	0	1 (1.5%)	0	0
HIF-1 signaling pathway	109	3 (2.8%)	2 (1.8%)	8 (7.3%)	0	0
Oxidative phosphorylation	134	0	0	2 (1.5%)	0	0
Pentose phosphate pathway	30	0	0	0	0	0
Pyruvate metabolism	47	0	0	0	0	0

Supplementary Table 3. SCPA analysis of the activity of 12 metabolic pathways in ectopic endometrium vs eutopic endometrium (by cell clusters).

Stromal

	Pathway	adjPval	qval	FC
1	HIF-1 signaling pathway	1.00E-52	7.2	-2.35
2	AMPK signaling pathway	2.37E-50	7.0	-3.29
3	Glutathione metabolism	3.55E-26	5.0	1.25
4	Glycolysis / Gluconeogenesis	3.71E-19	4.3	1.07
5	Pyruvate metabolism	1.82E-14	3.7	1.81
6	Fatty acid degradation	3.00E-13	3.5	1.80
7	Fatty acid elongation	1.90E-08	2.8	-0.30
8	ALA, ASP and GLU metabolism	5.55E-08	2.7	0.22
9	Oxidative phosphorylation	1.15E-06	2.4	-2.50
10	Pentose phosphate pathway	8.78E-01	0.2	0.31
11	Citrate cycle (TCA cycle)	1.00E+00	0.0	0.27
12	Fatty acid biosynthesis	1.00E+00	0.0	0.12

Endothelial

	Pathway	adjPval	qval	FC
1	HIF-1 signaling pathway	1.10E-57	7.5	3.19
2	AMPK signaling pathway	9.41E-51	7.1	0.26
3	Fatty acid elongation	8.12E-23	4.7	0.89
4	Glutathione metabolism	8.12E-23	4.7	-0.18
5	ALA, ASP and GLU metabolism	2.27E-13	3.6	0.05
6	Oxidative phosphorylation	4.80E-12	3.4	1.27
7	Pyruvate metabolism	8.64E-11	3.2	0.15
8	Citrate cycle (TCA cycle)	1.72E-08	2.8	-0.35
9	Glycolysis / Gluconeogenesis	1.90E-07	2.6	0.52
10	Fatty acid biosynthesis	1.54E-04	2.0	0.26
11	Fatty acid degradation	5.69E-04	1.8	-0.31
12	Pentose phosphate pathway	4.40E-02	1.2	0.20

Immune

	Pathway	adjPval	qval	FC
1	HIF-1 signaling pathway	1.91E-10	3.1	-0.80
2	AMPK signaling pathway	2.80E-01	0.7	-0.08
3	ALA, ASP and GLU metabolism	1.00E+00	0.0	0.04
4	Citrate cycle (TCA cycle)	1.00E+00	0.0	-0.43
5	Fatty acid biosynthesis	1.00E+00	0.0	-0.09
6	Fatty acid degradation	1.00E+00	0.0	-0.43
7	Fatty acid elongation	1.00E+00	0.0	-0.29
8	Glutathione metabolism	1.00E+00	0.0	-0.53
9	Glycolysis / Gluconeogenesis	1.00E+00	0.0	-0.43
10	Oxidative phosphorylation	1.00E+00	0.0	-3.08
11	Pentose phosphate pathway	1.00E+00	0.0	-0.16
12	Pyruvate metabolism	1.00E+00	0.0	-0.39

Perivascular

	Pathway	adjPval	qval	FC

1	HIF-1 signaling pathway	1.67E-69	8.3	-3.32
2	AMPK signaling pathway	2.17E-56	7.5	-3.52
3	Glutathione metabolism	1.56E-51	7.1	0.06
4	Oxidative phosphorylation	9.18E-29	5.3	7.12
5	Glycolysis / Gluconeogenesis	2.15E-16	4.0	0.21
6	Pentose phosphate pathway	1.59E-11	3.3	-0.29
7	Pyruvate metabolism	5.59E-11	3.2	0.38
8	Fatty acid elongation	1.91E-10	3.1	-0.08
9	Fatty acid degradation	6.32E-10	3.0	0.72
10	Citrate cycle (TCA cycle)	1.15E-06	2.4	0.02
11	ALA, ASP and GLU metabolism	3.76E-03	1.6	-0.21
12	Fatty acid biosynthesis	1.14E-01	1.0	-0.52

Macrophage

	Pathway	adjPval	qval	FC
1	HIF-1 signaling pathway	5.70E-10	3.0	2.49
2	AMPK signaling pathway	5.12E-05	2.1	1.95
3	Fatty acid biosynthesis	5.87E-02	1.1	1.15
4	Fatty acid degradation	9.11E-02	1.0	0.78
5	Glutathione metabolism	9.11E-02	1.0	-0.58
6	Oxidative phosphorylation	4.26E-01	0.6	-1.80
7	ALA, ASP and GLU metabolism	8.11E-01	0.3	0.31
8	Pyruvate metabolism	8.11E-01	0.3	-0.54
9	Citrate cycle (TCA cycle)	1.00E+00	0.0	-0.48
10	Fatty acid elongation	1.00E+00	0.0	-0.40
11	Glycolysis / Gluconeogenesis	1.00E+00	0.0	-0.23
12	Pentose phosphate pathway	1.00E+00	0.0	0.18

Unknown

	Pathway	adjPval	qval	FC
1	AMPK signaling pathway	1.82E-05	2.2	-7.62
2	HIF-1 signaling pathway	1.82E-05	2.2	-6.14
3	Glutathione metabolism	8.22E-04	1.8	-2.80
4	Pyruvate metabolism	6.81E-02	1.1	-2.83
5	Glycolysis / Gluconeogenesis	5.61E-01	0.5	-3.24
6	ALA, ASP and GLU metabolism	1.00E+00	0.0	-0.66
7	Citrate cycle (TCA cycle)	1.00E+00	0.0	-1.68
8	Fatty acid biosynthesis	1.00E+00	0.0	-0.49
9	Fatty acid degradation	1.00E+00	0.0	-1.84
10	Fatty acid elongation	1.00E+00	0.0	-1.13
11	Oxidative phosphorylation	1.00E+00	0.0	-15.65
12	Pentose phosphate pathway	1.00E+00	0.0	-0.81

B-cell

	Pathway	adjPval	qval	FC
1	HIF-1 signaling pathway	3.57E-02	1.2	0.87
2	AMPK signaling pathway	1.42E-01	0.9	-0.77
3	ALA, ASP and GLU metabolism	1.00E+00	0.0	-0.50

4	Citrate cycle (TCA cycle)	1.00E+00	0.0	0.07
5	Fatty acid biosynthesis	1.00E+00	0.0	0.25
6	Fatty acid degradation	1.00E+00	0.0	0.26
7	Fatty acid elongation	1.00E+00	0.0	0.02
8	Glutathione metabolism	1.00E+00	0.0	0.24
9	Glycolysis / Gluconeogenesis	1.00E+00	0.0	0.03
10	Oxidative phosphorylation	1.00E+00	0.0	-2.26
11	Pentose phosphate pathway	1.00E+00	0.0	-0.12
12	Pyruvate metabolism	1.00E+00	0.0	0.50

Epithelial

	Pathway	adjPval	qval	FC
1	HIF-1 signaling pathway	7.09E-11	3.2	3.41
2	AMPK signaling pathway	4.67E-05	2.1	-4.02
3	ALA, ASP and GLU metabolism	4.67E-05	2.1	0.99
4	Fatty acid elongation	3.72E-02	1.2	1.57
5	Glutathione metabolism	1.85E-01	0.9	1.31
6	Oxidative phosphorylation	1.85E-01	0.9	11.47
7	Citrate cycle (TCA cycle)	1.00E+00	0.0	0.92
8	Fatty acid biosynthesis	1.00E+00	0.0	-0.45
9	Fatty acid degradation	1.00E+00	0.0	0.89
10	Glycolysis / Gluconeogenesis	1.00E+00	0.0	3.14
11	Pentose phosphate pathway	1.00E+00	0.0	0.50
12	Pyruvate metabolism	1.00E+00	0.0	1.88

Cycling Stromal

	Pathway	adjPval	qval	FC
1	HIF-1 signaling pathway	1.05E-14	3.7	9.10
2	AMPK signaling pathway	1.00E+00	0.0	0.92
3	ALA, ASP and GLU metabolism	1.00E+00	0.0	0.13
4	Citrate cycle (TCA cycle)	1.00E+00	0.0	0.53
5	Fatty acid biosynthesis	1.00E+00	0.0	0.18
6	Fatty acid degradation	1.00E+00	0.0	3.31
7	Fatty acid elongation	1.00E+00	0.0	2.16
8	Glutathione metabolism	1.00E+00	0.0	0.26
9	Glycolysis / Gluconeogenesis	1.00E+00	0.0	3.48
10	Oxidative phosphorylation	1.00E+00	0.0	0.25
11	Pentose phosphate pathway	1.00E+00	0.0	0.42
12	Pyruvate metabolism	1.00E+00	0.0	0.82

Supplementary Table 4. Statistically significant differentially expressed genes of metabolic pathways between eutopic endometrium of controls (women without endometriosis) compared to eutopic endometrium of women with endometriosis from external dataset (Huang et al. 2023).

Perivascular cell type

gene	log2FoldChange	pvalue	padj	gene name	KEGG pathway	Pathway
VEGFA	-2.28	1.07E-08	0.000105	vascular endothelial growth factor A	hsa04066	HIF-1 signaling pathway - Homo sapiens (human)

Stromal cell type

gene	log2FoldChange	pvalue	padj	gene name	KEGG pathway	Pathway
IRS2	-1.52	1.94E-05	0.011439	insulin receptor substrate 2	hsa04152	AMPK signaling pathway - Homo sapiens (human)
LDHA	-1.14	3.65E-05	0.018793	lactate dehydrogenase A	hsa00010,hsa04066,hsa00620	Glycolysis / Gluconeogenesis - Homo sapiens (human),HIF-1 signaling pathway - Homo sapiens (human),Pyruvate metabolism - Homo sapiens (human)
PFKFB3	-1.49	8.2E-05	0.034727	6-phosphofructo-2-kinase/fructose-2,6-biphosphatase 3	hsa04152,hsa04066	AMPK signaling pathway - Homo sapiens (human),HIF-1 signaling pathway - Homo sapiens (human)

Endothelial cell type

gene	log2FoldChange	pvalue	padj	gene name	KEGG pathway	Pathway
NOS3	-1.28	1.21E-05	0.006569	nitric oxide synthase 3	hsa04066	HIF-1 signaling pathway - Homo sapiens (human)
SERPINE1	0.00	6.25E-05	0.024682	serpin family E member 1	hsa04066	HIF-1 signaling pathway - Homo sapiens (human)
IGF1	1.93	7.16E-05	0.027028	insulin like growth factor 1	hsa04152,hsa04066	AMPK signaling pathway - Homo sapiens (human),HIF-1 signaling pathway - Homo sapiens (human)

Supplementary Table 5. Total cell counts and percentages for perivascular, stromal and endothelial clusters in ectopic endometrium (EcE) and eutopic endometrium (EuE) by cell cycle phases. Fisher's exact test was used to compare cell cluster proportions between EcE and EuE for each cell cycle phase.

G1 phase	Perivascular, N (%)	Stromal, N (%)	Endothelial, N (%)
EcE	981 (50%)	592 (47%)	593 (36%)
EuE	355 (67%)	3201 (75%)	204 (61%)
Fisher's exact (P value)	2.78E-134	<4.58E-249	9.68E-88

G2/M phase	Perivascular, N (%)	Stromal, N (%)	Endothelial, N (%)
EcE	490 (25%)	373 (30%)	341 (21%)
EuE	62 (12%)	370 (9%)	54 (16%)
Fisher's exact (P value)	3.18E-165	0.92	2.83E-102

S phase	Perivascular, N (%)	Stromal, N (%)	Endothelial, N (%)
EcE	488 (25%)	283 (23%)	696 (43%)
EuE	110 (21%)	713 (16%)	77 (23%)
Fisher's exact (P value)	1.28E-113	4.81E-85	4.58E-249

Supplementary Table 6. Gene markers used for cell cluster annotation. The cluster names are

Cluster name	Stromal cells	Stromal fibroblasts		Cycling stromal cells
Cluster ID	0, 2	5	11	15
Gene markers	IGF1	IGF1	IGF1	IGF1
	ALDH1A2	ALDH1A2	ALDH1A2	ALDH1A2
	PAMR1	PAMR1	PAMR1	PAMR1
	MMP11	MMP11	MMP11	MMP11
	DPP6	LUM	SFRP1	SFRP4
	WT1	OGN	ACTA2	MKI67
		PDGFRA	CNN1	TOP2A
			LONRF2	TYMS
Merged clusters	Stromal			Cycling stromal

Cluster name	Endothelial vein cells	Endothelial artery cells	B cells
Cluster ID	1	16	12
Gene markers	EMCN	EMCN	BANK1
	ADGRL4	ADGRL4	CD79A
	VWF	VWF	CD37
	PECAM1	PECAM1	MS4A1
	CD34	CD34	
	ACKR1	SEMA3G	
	PLVAP	GJA5	
Merged clusters	Endothelial		B cell

Cluster name	Natural killer cells	T cells	Innate lymphoid cells	Macrophages
Cluster ID	3	4	17	8
Gene markers	PTPRC	PTPRC	PTPRC	PLEK
	STAT4	STAT4	STAT4	LYZ
	CD69	CD69	CD69	AIF1
	CCL5	IL7R	IL7R	IL1B
	AOAH	CD8A	KIT	MS4A6A
	GZMA		CXCR4	CD68
	NKG7			CD14
Merged clusters	Immune			Macrophage

Cluster name	Perivascular cells (RGS5)	Perivascular cells (STEAP4)	Perivascular cells (MYH11)	Perivascular cells (GUCY1A2)
Cluster ID	6	7	9	14
Gene markers	RGS5	STEAP4	MYH11	GUCY1A2
	NOTCH3	NOTCH3	NOTCH3	NOTCH3
	PDE3A	PDE3A	PDE3A	PDE3A
	MYH11	MYH11	NTRK3	MYH11
	TBX2	ABCC9	NTRK2	OLFML2B
	CDH6	GGT5	FHL5	AFAP1L2
	NDUFA4L2			
Merged clusters	Perivascular			

Cluster name	Epithelial cells	Unknown cells
Cluster ID	13	10
Gene markers	RHEX<br>EYA2<br>EPCAM<br>PROM1<br>ENPP3<br>FGFR2<br>CDH1<br>SOX9<br>PIFO<br>WFDC2	*No specific marker gene set was identified
Merged clusters	Epithelial	Unknown

Supplementary Table 7. A list of metabolic pathways obtained from KEGG pathway database, Homo sapiens (human).

Pathway name	Pathway identifier	No. of genes
Alanine, aspartate and glutamate metabolism	hsa00250	37
AMPK signaling pathway	hsa04152	121
Citrate cycle (TCA cycle)	hsa00020	30
Fatty acid biosynthesis	hsa00061	18
Fatty acid degradation	hsa00071	43
Fatty acid elongation	hsa00062	27
Glutathione metabolism	hsa00480	57
Glycolysis / Gluconeogenesis	hsa00010	67
HIF-1 signaling pathway	hsa04066	109
Oxidative phosphorylation	hsa00190	134
Pentose phosphate pathway	hsa00030	30
Pyruvate metabolism	hsa00620	47

Supplementary Table 8. A list of genes from steroidogenesis pathway.

Gene	Ensembl gene ID
ESR1	ENSG00000091831
ESR2	ENSG00000140009
AR	ENSG00000169083
GR	ENSG00000113580
AKR1C1	ENSG00000187134
AKR1C2	ENSG00000151632
AKR1C4	ENSG00000198610
AKR1D1	ENSG00000122787
ARO	ENSG00000137869
CYP11A1	ENSG00000140459
CYP11B1	ENSG00000160882
CYP11B2	ENSG00000179142
CYP17A1	ENSG00000148795
CYP19A1	ENSG00000137869
CYP21A2	ENSG00000231852
DHRS11	ENSG00000278535
HSD11B1	ENSG00000117594
HSD11B2	ENSG00000176387
HSD17B1	ENSG00000108786
HSD17B10	ENSG00000072506
HSD17B11	ENSG00000198189
HSD17B13	ENSG00000170509
HSD17B14	ENSG00000087076
HSD17B2	ENSG00000086696
HSD17B3	ENSG00000130948
HSD17B4	ENSG00000133835
HSD17B5	ENSG00000196139
HSD17B6	ENSG00000025423
HSD17B7	ENSG00000132196
HSD17B8	ENSG00000204228
HSD17B9	ENSG00000135437
HSD3B1	ENSG00000203857
HSD3B2	ENSG00000203859
RDH11 (HAS15)	ENSG00000072042
SRD5A1	ENSG00000145545
SRD5A2	ENSG00000277893
SRD5A3	ENSG00000128039
STAR	ENSG00000147465
STS	ENSG00000101846
SULT1A1	ENSG00000196502
SULT1A2	ENSG00000197165
SULT1A3	ENSG00000261052
SULT1B1	ENSG00000173597
SULT1E1	ENSG00000109193
SULT2A1	ENSG00000105398
SULT2B1	ENSG00000088002
PGR	ENSG00000082175
