# Supplementary material for: Endometriotic lesions exhibit distinct metabolic signature compared to paired eutopic endometrium at the single-cell level
Source: Commun Biol. 2024 Aug 21;7:1026. doi: 10.1038/s42003-024-06713-5 (PMC11339455; doi:10.1038/s42003-024-06713-5)
Supplement: Supplementary file 3 — Description of Additional Supplementary Files [file 42003_2024_6713_MOESM3_ESM.pdf]

Description of Additional Supplementary Files

File name: Supplementary Data 1

Description: The mean scores of the activity of transcription factors (TF) in stromal, perivascular and endothelial cell populations of eutopic endometrium (EuE) and ectopic endometrium (EcE).

File name: Supplementary Data 2

Description: Statistically significant differentially regulated genes of metabolic pathways between ectopic endometrium and eutopic endometrium. NS - statistically non significant value (padj < 0.05)

File name: Supplementary Data 3

Description: The source data (raw transcriptomic data) behind the tables and graphs in the paper deposited at GEO repository.
