# Supplementary material for: Endometriotic lesions exhibit distinct metabolic signature compared to paired eutopic endometrium at the single-cell level
Source: Commun Biol. 2024 Aug 21;7:1026. doi: 10.1038/s42003-024-06713-5 (PMC11339455; doi:10.1038/s42003-024-06713-5)
Supplement: Supplementary file 6 — Reporting Summary [file 42003_2024_6713_MOESM6_ESM.pdf]

nature portfolio

Corresponding author(s): Andres Salumets
Last updated by author(s): Jul 18, 2024

Reporting Summary

Nature Portfolio wishes to improve the reproducibility of the work that we publish. This form provides structure for consistency and transparency in reporting. For further information on Nature Portfolio policies, see our Editorial Policies and the Editorial Policy Checklist.

Statistics

For all statistical analyses, confirm that the following items are present in the figure legend, table legend, main text, or Methods section.

n/a	Confirmed	
☐	☒	The exact sample size (*n*) for each experimental group/condition, given as a discrete number and unit of measurement
☐	☒	A statement on whether measurements were taken from distinct samples or whether the same sample was measured repeatedly
☐	☒	The statistical test(s) used AND whether they are one- or two-sided<br>*Only common tests should be described solely by name; describe more complex techniques in the Methods section.*
☒	☐	A description of all covariates tested
☐	☒	A description of any assumptions or corrections, such as tests of normality and adjustment for multiple comparisons
☒	☐	A full description of the statistical parameters including central tendency (e.g. means) or other basic estimates (e.g. regression coefficient) AND variation (e.g. standard deviation) or associated estimates of uncertainty (e.g. confidence intervals)
☐	☒	For null hypothesis testing, the test statistic (e.g. *F*, *t*, *r*) with confidence intervals, effect sizes, degrees of freedom and *P* value noted<br>*Give P values as exact values whenever suitable.*
☒	☐	For Bayesian analysis, information on the choice of priors and Markov chain Monte Carlo settings
☒	☐	For hierarchical and complex designs, identification of the appropriate level for tests and full reporting of outcomes
☒	☐	Estimates of effect sizes (e.g. Cohen's *d*, Pearson's *r*), indicating how they were calculated

Our web collection on statistics for biologists contains articles on many of the points above.

Software and code

Policy information about availability of computer code

Data collection	NA
Data analysis	All scripts and details on processing steps were deposited in GitHub and are available at: https://github.com/ankita16lawarde/scRNA-Seq_endometriosis.

For manuscripts utilizing custom algorithms or software that are central to the research but not yet described in published literature, software must be made available to editors and reviewers. We strongly encourage code deposition in a community repository (e.g. GitHub). See the Nature Portfolio guidelines for submitting code & software for further information.

Data

Policy information about availability of data

All manuscripts must include a data availability statement. This statement should provide the following information, where applicable:

- Accession codes, unique identifiers, or web links for publicly available datasets
- A description of any restrictions on data availability
- For clinical datasets or third party data, please ensure that the statement adheres to our policy

The raw transcriptomic data were deposited at the GEO repository and are available under the GEO accession number GSE247695.

Research involving human participants, their data, or biological material

Policy information about studies with human participants or human data. See also policy information about sex, gender (identity/presentation), and sexual orientation and race, ethnicity and racism.

Reporting on sex and gender	The data obtained and the findings are applicable to the female sex due to the nature of the disease (endometriosis) and its occurrence only in women's reproductive organs.
Reporting on race, ethnicity, or other socially relevant groupings	The women recruited to this study were from Estonian population.
Population characteristics	All four women were in their follicular phase of the menstrual cycle (days 7 - 11), 33 ± 6.4 years old (mean ± standard deviation), with normal body mass index of 21 ± 1.8 kg/m2, and had not received any hormonal treatments for at least 3 months prior to the time of laparoscopy and sample collection. According to the revised American Society for Reproductive Medicine classification system, three women had a minimal-mild endometriosis and one woman had a moderate-severe endometriosis.
Recruitment	Both, paired eutopic endometrium and ectopic endometrium samples were collected from four women with endometriosis undergoing laparoscopic surgery at the Tartu University Hospital (Tartu, Estonia).
Ethics oversight	The study was approved by the Research Ethics Committee of the University of Tartu, Estonia (No 333/T-6), and written informed consent was obtained from all participants.

Note that full information on the approval of the study protocol must also be provided in the manuscript.

Field-specific reporting

Please select the one below that is the best fit for your research. If you are not sure, read the appropriate sections before making your selection.

☒ Life sciences ☐ Behavioural & social sciences ☐ Ecological, evolutionary & environmental sciences

For a reference copy of the document with all sections, see nature.com/documents/nr-reporting-summary-flat.pdf

Life sciences study design

All studies must disclose on these points even when the disclosure is negative.

Sample size	Sample size was limited to eight samples (paired eutopic endometrium and ectopic endometrium) from four women with endometriosis (four biological replicates for each experimental group). Considering the power of the technique analysing single cell transcriptome of different cell populations in each sample, this sample size is statistically justified.
Data exclusions	Only data from women with confirmed endometriosis and not receiving hormonal treatment for at least three months were included in this study.
Replication	The findings can be replicated by following experimental approach and bioinformatic analysis used in this study and described in details in the manuscript. Within the revision, we performed the analyses of cell clusters and metabolic and steroidogenic pathways using external dataset (Huang et al. 2023)
Randomization	The samples collected from four women with confirmed endometriosis were paired (eutopic endometrium and corresponding ectopic endometrium), and grouped by the tissue type histologically evaluated and confirmed by a pathologist.
Blinding	The blinding was not relevant in this study as bioinformatic analysis of the data was applied.

Reporting for specific materials, systems and methods

We require information from authors about some types of materials, experimental systems and methods used in many studies. Here, indicate whether each material, system or method listed is relevant to your study. If you are not sure if a list item applies to your research, read the appropriate section before selecting a response.

Materials & experimental systems

n/a	Involved in the study
☒	☐ Antibodies
☒	☐ Eukaryotic cell lines
☒	☐ Palaeontology and archaeology
☒	☐ Animals and other organisms
☒	☐ Clinical data
☒	☐ Dual use research of concern
☒	☐ Plants

Methods

n/a	Involved in the study
☒	☐ ChIP-seq
☒	☐ Flow cytometry
☒	☐ MRI-based neuroimaging

Plants

Seed stocks	NA
Novel plant genotypes	NA
Authentication	NA
